# Results from the Adverse Event Sedation Reporting Tool: A Global Anthology of 7952 Records Derived from >160,000 Procedural Sedation Encounters

**DOI:** 10.3390/jcm8122087

**Published:** 2019-12-01

**Authors:** Keira P. Mason, Mark G. Roback, David Chrisp, Nicole Sturzenbaum, Lee Freeman, David Gozal, Firoz Vellani, David Cavanaugh, Steven M. Green

**Affiliations:** 1 Harvard Medical School, Boston Children’s Hospital, Dept. of Anesthesiology, Critical Care and Pain Medicine, 300 Longwood Ave., Boston, MA 02115, USA; 2 University of Colorado School of Medicine, Section Head, Emergency Medicine Colorado Children’s Hospital, Aurora, CO 80045, USA; Mark.Roback@childrenscolorado.org; 3 Tauranga OMS, PO. BOX 15557, 3144 Tauranga, New Zealand; david@taurangaoms.co.nz; 4 Toothbeary Richmond, 358a Richmond Road, East Twickenham TW1 2DU, UK; nicole@toothbeary.co.uk; 5 Summit Anesthesia Services, 1591 Ridge West Dr., Windsor, CO 80550, USA; lwf1970@gmail.com; 6 Department of Anesthesiology and Critical Care Medicine, Hadassah University Hospital, 91120 Jerusalem, Israel; davidgozal@yahoo.com; 7 PO Box 792, Cherrybrook, NSW 2126, Australia; firoz04@yahoo.com; 8 Boston Biostatistical Consulting, North Reading, MA 01864, USA; dmcav6@gmail.com; 9 The Department of Emergency Medicine, Loma Linda University Medical Center, Loma Linda, CA 92354, USA; steve@stevegreenmd.com

**Keywords:** adults, pediatrics, safety, sedation, children

## Abstract

*Background*: The incidence of sedation-related adverse events, inclusive of both adults and children, administered by multiple specialty providers from different countries and venues, using standardized definitions, has never been reported on an international level. We are reporting the outcome data of the adverse event sedation reporting tool as an important step toward a more complete risk assessment of sedation-related morbidity, mortality, and etiology. The analysis of the AE sedation reporting data include descriptive measures to evaluate the characteristics of the provider, the patient, sedations performed, adverse events, interventions, and outcomes. The primary outcome was the rate and nature of adverse events. Between 12/14/2010 and 12/11/2018 there were 7952 sedations, from an estimated total of 164,114 sedations administered, of which 622 were reported as adverse events. The mean age of the entire patient population is 33.0 years (0.02–98.7). The providers represented 39 countries across six continents. Oxygen desaturation (75%–90%) for <60 s is the most prevalent adverse event with a rate of 7.8 per 10,000, followed by airway obstruction at a rate of 5.42 per 10,000. Apnea occurred at a rate of 4.75 per 10,000. Significant predictors of adverse events are ≥ ASA score III (*p* = 0.0003), procedure time (6:00 pm–12:00 am: *p* < 0.0001, 12:00–6:00 am: *p* = 0.0003), and non-hospital location (*p* < 0.0001). The AE sedation reporting tool has demonstrated that the majority of adverse events in children and adults who receive procedural sedation from multi-specialists internationally required minor interventions and had outcomes of minor risk.

## 1. Introduction

Procedural sedation, for both adults and children, encompasses a wide range of patients, procedures, venues, and specialty providers. The collecting of sedation outcomes can be challenging, as it requires vigilant need to identify, report, and document multiple identifiers. To date, there is a paucity of large scale reports of sedation outcome. The large scale studies in the literature are generally limited to a single patient population (adult, pediatric), sedation provider (pediatrician, dentist, emergency medicine, anesthesia, nursing), sedation technique (medication, route, delivery method), venue (academic or non-academic setting, dental office, hospital based, non-hospital based), procedure (endoscopy, magnetic resonance imaging), and country (United States, Europe, Asia) [1,2,3,4,5,6,7,8,9,10,11,12,13,14,15,16,17,18,19,20,21]. The largest scale studies to date have reported outcomes from adults in either Europe or the United States who received procedural sedation for gastrointestinal endoscopic procedures [13,22,23,24]. 

This report is the first to present the incidence of adverse events, collectively for both adults and children, for sedation delivered by multiple specialty providers performing a wide range of procedures in different countries and venues. The adverse event (AE) sedation reporting tool was published online in 2011 (www.AESedationReporting.com) and published as a manuscript in 2012, as the first tool in existence which would standardize the reporting and tracking of all sedation-related adverse events [25]. It has since been accepted and adopted as a means for sedation providers worldwide to objectively identify sedation-related outcomes [26,27,28,29,30,31,32,33,34]. As a web-based, free-of-charge tool for which each user has his own password to access and log-in his/her data which is void of HIPAA identifiers, the AE sedation reporting tool is a valuable means to collect and track data. By identifying and analyzing important demographics and outcomes from each sedation encounter, this tool can provide a benchmark for defining the occurrence and predictors of adverse events across continents, patient populations, procedures, venues, and providers. We are presenting all of the outcome data entered into the www.AESedationReporting.com website by providers worldwide. This database for sedation-outcome reporting supports the collection and sharing of data. The goal of this manuscript is to evaluate the adverse event sedation reporting data in order to determine whether there are any risk factors or predictors of adverse events. This is the first report of adverse event sedation data collected using this AE reporting tool, with international contributions of multi-specialists providing care of adult and pediatric populations. This report is an important first step toward a more complete risk assessment of sedation-related morbidity, mortality, and etiology that could direct efforts to further future safe sedation.

## 2. Materials and Methods 

**Figure 1:**
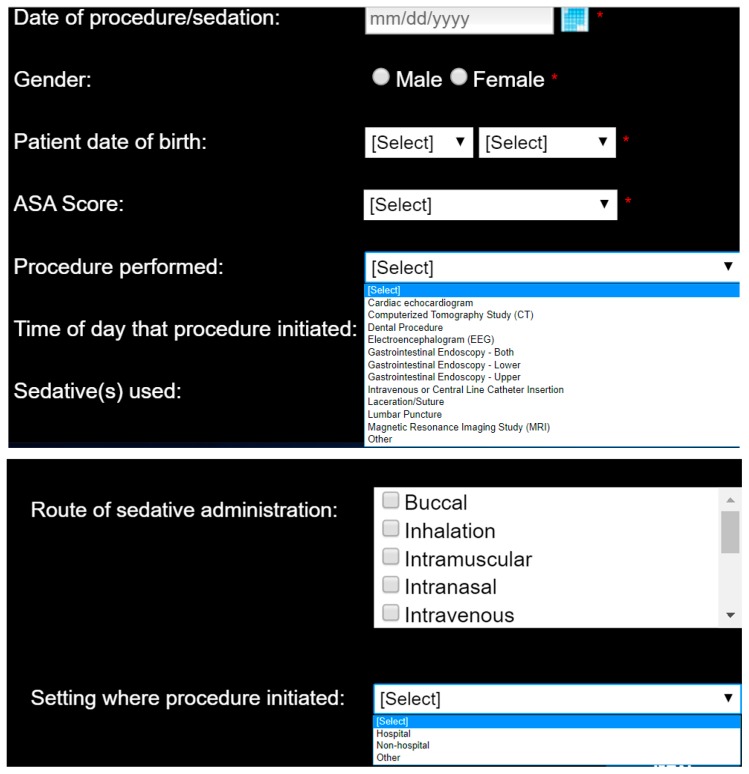
Sedation encounter demographics.

In 2012, an International Sedation Task Force (ISTF) comprised of physicians from 10 specialties to 11 countries (Australia, Brazil, China, Finland, Germany, Israel, Italy, Japan, South Africa, United Kingdom, and United States) presented the adverse event sedation reporting tool as a consensus document intended to standardize the definitions and terminology of sedation related adverse events [25]. The International Sedation Task Force comprised of members from all the continents and we specifically created a tool which would provide simple English and medical terms for the sedation providers to understand. To date we are under the impression that the tool has been translated in paper form to other languages but for online, it remains in English. The ISTF was comprised of sedation providers with established research, clinical experience, leadership, and expertise in the area of procedural sedation. Members were chosen with diverse backgrounds and from diverse specialties (dental medicine and anesthesia, emergency medicine, nursing, hospital medicine, intensive care medicine, anesthesia (physician and nurse), gastroenterology, pediatric medicine). The tool was intended to present a taxonomy of sedation outcomes which would be objective and reproducible. By adopting this taxonomy worldwide, particularly valuable in areas with neither organized sedation systems nor prior means of collecting and reporting their own outcomes, all sedation providers now have a means of tracking and sharing their outcomes through the AE sedation reporting tool database [26,29,31,34,35,36]. This tool is a repository of sedation-related data inputted from all sedation providers worldwide and is a means to report on sedation demographics and outcomes, with the objective of presenting a hierarchical structure to predict the occurrence, risk, and outcome of adverse events (Figure 1, Table 1, Table 2). 

### 2.1. Methods

The AE sedation reporting tool requires individual registration prior to utilization, to collect specifics that include the sedation provider’s first and last name, email address, specialty, place and type of practice, educational background, city and country of current practice, phone number and predominant patient population—adult, pediatric, or both. Every time that the registrant logs onto the website to input data, he/she is required to provide an estimate (or update) of the total number of sedations personally performed in a year. The tool consists of contiguous screens, through which the user progresses, each of which requires full completion prior to moving on to the next [25]. 

**Figure 2:**
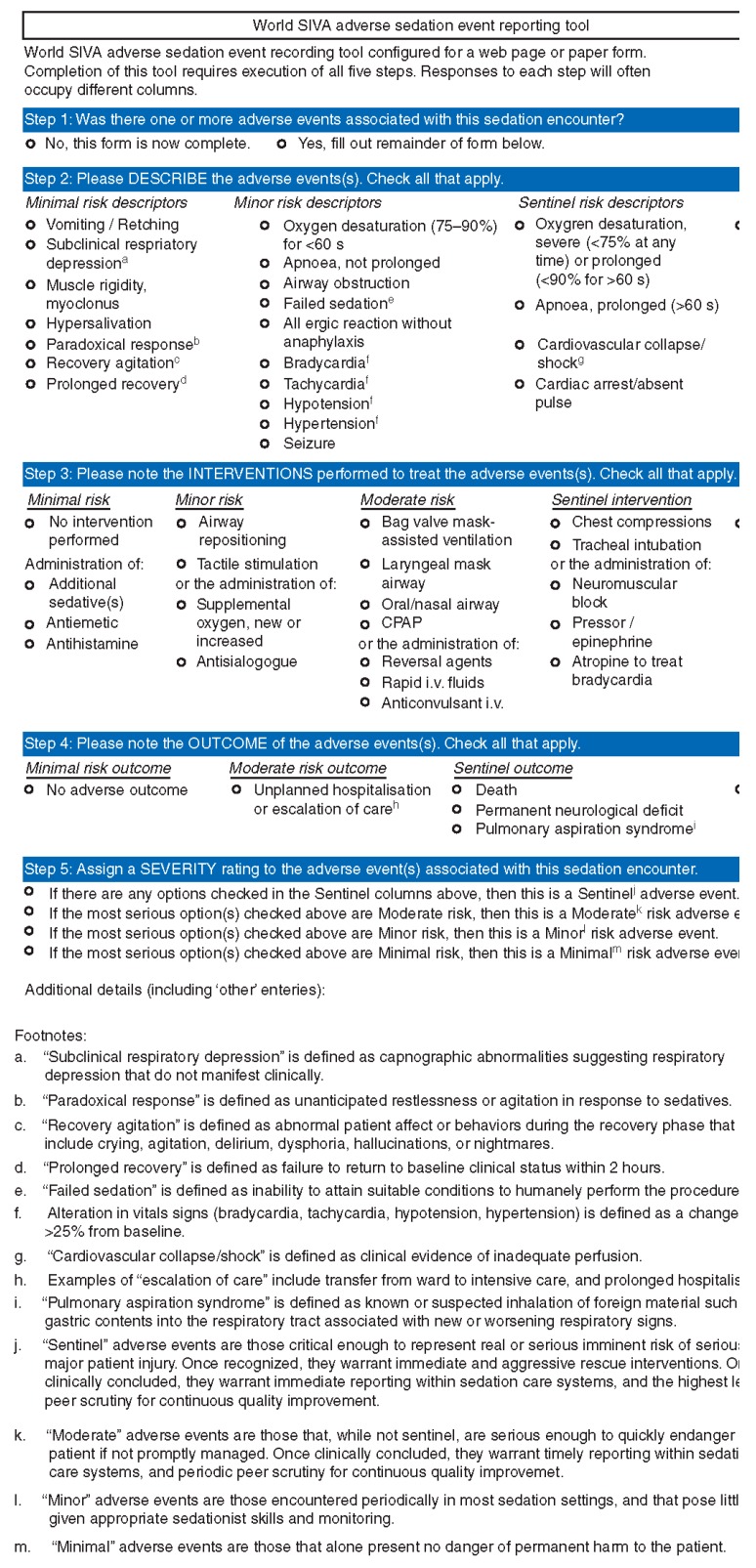
Adverse event sedation reporting tool [25].

The purpose is to identify the occurrence (or non-occurrence) of an adverse event, the urgency of the procedural sedation (emergent versus non-emergent), describe the adverse event using one or more objective, clearly defined descriptors and then in the event an intervention was needed, define what was performed (Figure 2). When entering data, each event and intervention is easily defined for the user, simply by hovering the mouse over the text. Each descriptor is categorized as minimal, minor, or sentinel. The definitions of the interventions performed are standardized and categorized by the risk level of the performed intervention, progressing from minimal (no intervention, antiemetics, antihistamines) to minor (airway repositioning), moderate (mask assisted ventilation), and sentinel (chest compressions). It is not possible to identify which intervention was related to which adverse event when multiple events occurred. Each screen and query must be answered for the user to progress through the reporting tool.

Outcome measures are categorized as minimal (no adverse outcome), moderate (unplanned hospitalization or escalation of care), or sentinel (death, permanent neurological deficit, or pulmonary aspiration). There may be occasions in which there may be more than one outcome, each with different severity (moderate and sentinel, for example). In this circumstance, the overall severity of the adverse event is assigned a rating (minimal, moderate, sentinel) based on the most severe outcome.

### 2.2. Statistical Analysis

The analysis of the AE sedation reporting data included descriptive measures to evaluate the characteristics of the provider, the patients, sedations performed, interventions, outcomes, and their adverse events. The primary outcome was the rate of adverse events. The secondary outcome was to determine predictor variables of adverse events from sedation.

Incidence rates of adverse events were calculated using the number of events and the estimated number of sedations per year by the provider. Their cumulative sum will be representative of the entire sample of sedation related adverse and non-adverse events. A stratified random re-sampling simulation of non-adverse events based on the distribution of non-adverse events reported was performed to enable further analyses. A multivariate logistic regression model was developed to identify predictors of adverse events. Predictor variables were chosen on the basis of the data (patient and provider) collected: American Society Anesthesiologist (ASA) score, age groupings, gender, time of procedure, and location of procedure. Odds ratios (ORs) and 95% confidence intervals (CIs) without adjustment for multiple comparisons were considered. *p*-value less than 0.05 were considered statistically significant. All statistical analysis was performed using SAS (version 9.4; SAS Institute, Inc., Cary, NC, USA). 

## 3. Results

The data for this study was collected from 12/14/2010 to 12/11/2018 and included 7952 sedation entries, of an estimated 164,114 total sedations performed over this time period. The premise is that from the estimated number of sedations performed each year, there were no adverse events that occurred in the remaining 156,162 cases that did not have data recorded (other than the 622 that were reported in the 7952 cases entered). All sedation entries had complete data points, as every screen must be completed in order for the user to continue data entry in the tool and save the data. 

The patient data presented in Table 3 represents their demographic information, with ASA physical status included. The mean age of the entire patient population is 33.0 years (0.02–98.7) years. The majority of adults who receive sedation are between 18 and 70 years of age, with a small minority (2.2%) over 80 years of age. The majority of children who receive sedation are between the ages of 3–12 years. Infants less than one year of age are less common, representing 0.6% of the population overall. The majority of patients (94.5%) are healthy (ASA 1 or 2), presenting for sedation for non-emergent procedure. Those procedures that are considered to be emergent, are most likely to be in healthy patients with ASA ≤2 (96.3%).

**Figure 3:**
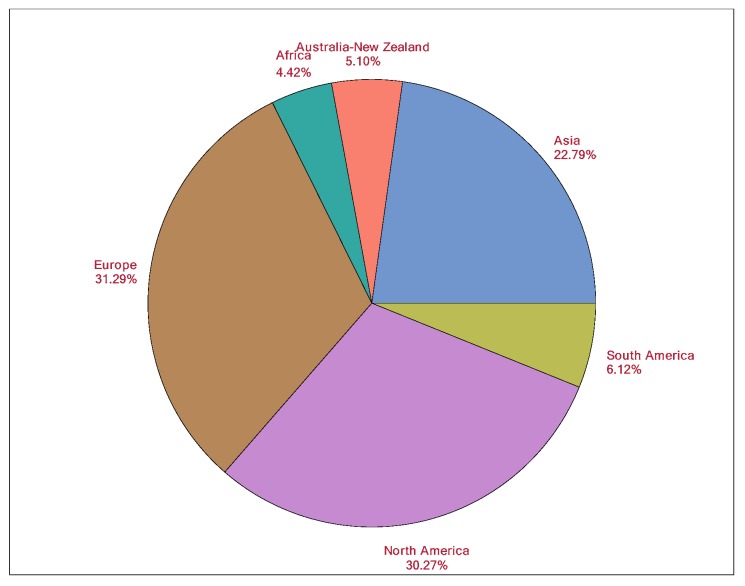
Legend: Distribution of provider’s continent of origin (*n* = 306).

The providers (users of the AE sedation-reporting tool) represent a mixture of physician (MD, DO, DMD) and non-physician professions from 39 countries across six continents (Figure 3). Physicians are the most common (79.1%) providers and include anesthesiologists, dental medicine, emergency medicine, hospital medicine, and pediatricians from 39 countries. There is a vast worldwide presence of AE Tool sedation providers. Europe represents 31.3% of the sedations, followed by North America (30.31%), and Asia (22.8%). Figure 3 shows a pie chart for the provider’s continent of origin, showing the diverse locations for the providers. The providers are from various backgrounds with respect to the academic or non-academic setting, hospital or out-of-hospital setting, and their patient population (adult and/or pediatric). Table 4 shows the country, place of practice, and academic or non-academic setting, where the sedations were performed. Within a country and place of practice, there is a range of one sedation performed to 3328 performed compromising of 0.01% and 42.2%, respectively. Most sedations are administered in a hospital-based setting (75.8%), with a slight propensity toward the academic (44.4%) versus non-academic setting (35.3%). Nearly a similar proportion of providers practice adult medicine (41.5%) compared to pediatric (<18 years old) medicine (36.9%). 21.6% of providers deliver sedation to both the adult and pediatric population. Interestingly, the distribution of adult versus pediatric sedations is nearly similar at 50.9% and 49.1%, respectively. The most frequently used sedative route is intravenous (87.2%), followed by inhalation (7.2%), and oral (2.4%).

For those patients who experienced adverse events, the summary statistics of their age and age group distribution are mean age is 32.1 (0.1–98.7) years. Our multivariable model presents 622 adverse events representing outcomes of a total of 164,114 sedations (Table 5). Reference groups are indicated for each predictor for which we can compare the other groups against. Several notable predictors were associated with higher risks: ASA score ≥ III or higher (OR 1.89), age groups 50–70 (OR 2.19) and 70–80 (OR 2.81), and procedure time occurring between 6:00 pm–12:00 am (OR 8.74) and 12:00–6:00 am (OR 5.86). Predictors of adverse events included ASA score ≥ III (*p* = 0.0003), age groups: 1–3 (P = 0.0181), 3–5 (*p* < 0.0001), 50–70 (*p* < 0.0001), 70–80 (*p* < 0.0001), procedure time of 12:00–6:00 pm (*p* = 0.0027), 6:00 pm–12:00 am (*p* < 0.0001), and 12:00–6:00 am (*p* = 0.0003), and non-hospital location (*p* < 0.0001).

**Figure 4:**
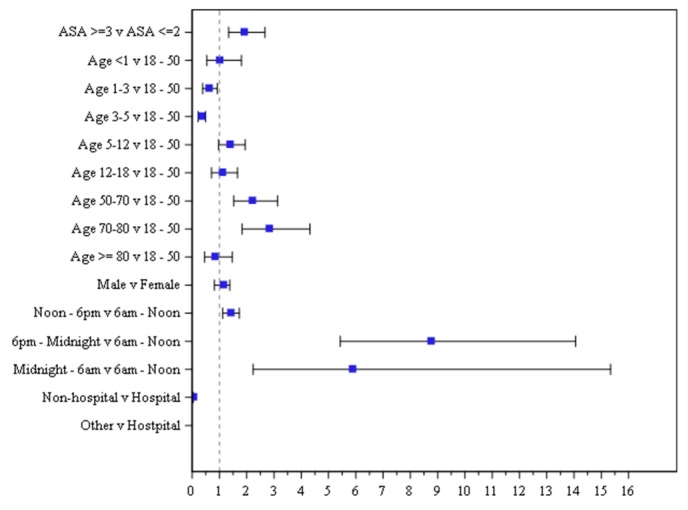
Forest plot depicting odds ratio and 95% confidence intervals.

With regards to significant predictors of age, the odds ratio show that age groups 1–2, 3–5, and ≥80 have lower odds of experiencing an AE than those 18–50 years old. The corresponding results of odds ratio and 95% CI is presented visually in Figure 4. The odds ratio is represented by the colored square and the 95% CIs are shown as lines for each individual predictor. The predictors that are significant are represented by having the odds ratio and 95% CI entirely outside of the vertical reference line of 1.

The identification of adverse events by risk descriptors is summarized in Table 6. This includes the risk of adverse events, the interventions performed, their outcomes, and the severity level. The most common sedation risk is at the moderate level (62.5%). The most common intervention and outcome risk is at the minor level, 59.5% and 93.4%, respectively. The most common severity is at the moderate level (61.5%). The sedation risks of adverse events are presented in Table 7. Of the 622 events, there were 94 (15.1%) sentinel events: Oxygen desaturation severe (<75% at any time) (*n* = 63), prolonged (>60 s) Apnea (*n* = 25), cardiovascular collapse / shock (*n* = 3), and cardiac arrest/absent pulse (*n* = 3). There were 389 (62.5%) cases of moderate events: Oxygen desaturation (75–90%) for <60 s (*n* = 128), and airway obstructions (*n* = 89). There were 139 (22.3%) cases of minor events: Vomiting/retching (*n* = 47). There were no cases with minimal risk. No deaths were reported. Sentinel events were analyzed separately to identify predictors of interest with similar results. All sedation providers of sentinel events were contacted to confirm the course and occurrence of events.

The intervention risks of adverse events are presented in Table 7. There were 768 total interventions associated with the adverse events, with 20 (2.6%) interventions identified as sentinel: Pressor/epinephrine (*n* = 6), atropine to treat bradycardia (*n* = 4), and chest compressions (*n* = 5). There were 124 (16.1%) moderate interventions: Oral/nasal airway (*n* = 51), bag valve mask (positive pressure ventilation) assisted ventilation (*n* = 49). There were 457 (59.5%) minor interventions: Airway repositioning (*n* = 183), supplemental (new or elevated concentration) oxygen (*n* = 147), and tactile stimulation (*n* = 120). There were 167 (21.7%) minimal interventions; 114 resolved spontaneously without intervention, 24 received additional sedative(s), and 22 received antiemetic. 

Adverse events and incidence rates per 10,000 with 95% CI are presented in Table 7. Oxygen desaturation was separated into categories based on degree and duration. Oxygen desaturation (75–90%) for <60 seconds and airway obstruction represented the most prevalent adverse events, occurring at a rate of 7.8 and 5.42 per 10,000, respectively, followed by apnea at 4.75 per 10,000. Severe (<75%) or prolonged (<90% for >60 s) oxygen desaturation occurred at a rate of 3.84 per 10,000.

All possible sedatives and combinations are available as options on the AE sedation reporting tool. The administration of sedatives (Propofol, Midazolam, Ketamine, Fentanyl, Alfentanil, Chloral hydrate, Clonidine, Dexmedetomidine), alone or in combination, were the most common choices and are summarized in Table 8. Propofol administered alone (18.7%), Fentanyl in combination with one other drug (11.9%), Alfentanil in combination with one other drug (11.0%), and Ketamine alone (8.8%) are the most common regimens. Other sedatives (Albuterol, Atropine, Dexamethasone, Diazepam, Diphenhydramine, Etomidate, Flumazenil, Fosphenytoin, Fospropofol, Meperidine, Morphine, Naloxone, Nitrous oxide, Remifentanil, Scopolamine, Sevoflurane, Sufentanil) used alone or in combination with other sedatives accounted for 13.76% of regimens. An association between sedatives and outcomes (sedation risk) was analyzed. There is a statistically significant association between sedatives and sedation risk, exact test *p*-value <0.0001. Specific associations between particular sedatives and risks was not able to be evaluated because of the broad spectrum of sedatives and combinations administered, via various routes. 

## 4. Discussion

Creating, implementing, and updating quality improvement data in a collaborative manner has become an important initiative of all published sedation guidelines worldwide. The collection of outcomes is not limited only to deep sedation or monitored anesthesia care, but also extends even to moderate depths of sedation. To date, all published outcome data has represented retrospective reviews of outcome data collected by specific groups of sedation providers, usually representing a single specialty, sedative agent, patient population (adults or children), and occasionally representing a group of varied specialists [2,6,22,37,38,39,40,41,42,43,44,45,46,47]. The data has almost always represented either pediatrics or adult patients, never representing sedation in both populations across continents.

This report has highlighted that with multi-specialty providers from a diverse background administering to the extreme spectrums of age for a variety of procedures, the overall risk of sedation is moderate. Almost 15% of all adverse events do not require any intervention. The majority of interventions, when required, are minor and involve airway repositioning most commonly for oxygen desaturation, airway obstruction, or apnea. When sentinel outcomes do occur, they are most likely to involve cardiac arrest, cardiovascular collapse, or oxygen desaturations <75%. Propofol and ketamine delivered as sole agents, are most common with intravenous route strongly favored. Most providers deliver either to adults or children, with only a minority delivering to both populations. In-hospital sedation in the academic setting tends to be more common. The increased risk with higher ASA status is consistent with prior published studies [5,13,48,49]. The findings of higher risks between 6 pm and 6 AM and for those sedations which were for emergent procedures have not previously been reported and warrant further investigation.

The limitations of our study are those shared by all quality assurance and database reports. The data represents only that of the sedation providers who input data. The sedationist makes his/her own determination whether to enter a sedation encounter into the adverse event reporting tool. Each provider may have his/her own threshold and criteria for entering data. As the website is open access and free-of-charge, it is not possible to surmise the rationale of different providers. The intent of the online tool is for sedation providers to tailor its use to his/her particular needs. The tool does not require HIPAA identifiers and the data is collected anonymously (users identity only known to the tool administrator (KPM), all with the intent to encourage “unintimidated” reporting. It is also possible that not every adverse event which occurred during this time period is captured. This is a limitation of all database reports. Particularly as sedation is not commonly captured by electronic medical records, often performed by multi-specialists, the data is unavailable to be extricated electronically. In under-developed areas, electronic medical records are often unavailable and non-existent. Even the most sophisticated of electronic records are unable to accurately capture all events: They may be able to capture physiological vital signs, but fail to document the interventions performed and never are able to record the outcomes.

This database is completely founded on clinician self-report and, thus, risks under-reporting of adverse events. There are multiple etiologies for under-reporting: Sedationists may not want to appear to be “poorly performing” by reporting adverse events, some institutions may have policies in place that may generate “punitive” reactions to documented adverse events (e.g., initiating root cause analysis proceedings) and others may simply forget to document the adverse event in the course of providing clinical care because it is not routine practice to use this tool on a regular basis. Thus, the self-reporting of the adverse event reporting tool differentiates this database from others which implement more comprehensive approaches to maximize capture and review of all data. For example, some databases require entry of consecutive cases, ongoing database surveillance, manual audits of randomly selected charts, automated validation checks of their data, and review of interrater reliability prior to data collection and on an ongoing basis.

Brief events (oxygen desaturation, hypotension, tachycardia, bradycardia, decreased respiratory rate) may not be captured even by electronic medical records, depending on how frequently these events are recorded. Typically, vital signs and physiological parameters are reported every 5 min. Thus, occurrences within this 5 min window may often go unreported, even electronically. As with any database collecting system, there is a tendency for the reporters to be a self-selected group of individuals who are likely to have some special interest in sedation. Thus, it is possible that these outcomes may not mirror the clinical practice of sedation as a whole.

The advantage of the AE reporting tool is that it is the only online, open access, and free-of-charge site that allows providers to not only input their data but also be able to follow and track their own outcomes. Any provider with access to the internet has the ability to report and collect their sedation outcomes. Free of HIPAA identifiers, the user is able to collect data but also still be able to query a particular record if some of the basic specifics of the procedure or event are known. This ability makes the AE tool unique and invaluable to providers, particularly those who lack an organized infrastructure needed to record and collect data as obliged by the Joint Commission and International Joint Commission. The AE tool importantly ensures that all data points are collected, because the user is unable to proceed to the following queries and to complete the data input, unless all data is entered. As adverse events tend to be memorable, as are the associated interventions and outcomes, we believe and assume that those who enter the data have a clear recollection of the event and, often, probably are referring to the patient’s medical records as demographics are included (age, sedatives, routes of administration, etc.). The database is unusual because a significant proportion of the data includes dental sedation.

The adverse event sedation reporting tool as well as this report, represents the monolithic effort of multi-specialty providers from six continents to identify and classify their sedation related outcomes, using definitions that have been standardized and adopted for clinical and research purposes. Our report of an increased risk of adverse events in ASA three or higher, evening sedations, the middle age and elderly adult, and out of hospital procedures, are revealing first steps to guide future studies of sedation-outcome, as well as the design of sedation services.

## Figures and Tables

**Table 1:** Sedative options on adverse event sedation reporting tool website.

Sedative(s) Used
Alfentanil (Alfenta, Rapifen)
Atropine
Chloral hydrate
Clonidine
Dexamethasone
Dexmedetomidine (Precedex)
Diazepam (Valium, Antenex)
Diphenhydramine (Benadryl, Dimedrol, Daedalon)
Diphenhydramine (Benadryl, DPH, DHM, Dimedrol, Daedalon)
Epinephrine (adrenaline)
Etomidate (Amidate)
Fentanyl (Fentanil, Sublimaze, Fentora, Onsolis, Instanyl, Asbtral)
Flumazenil (reversal agent for benzodiazepines—flumazepil, Anexate, Lanexat, Mazicon, Romazicon, Anexate)
Fosphenytoin (usually used to treat seizures—Cerebyx, Prodilantin)
Fospropofol (Lusedra)
Ketamine (Ketanest, Ketanest, Ketaset, Ketalar)
Ketamine (Ketanest, Ketanest, Ketaset, Ketala)
Lidocaine (a local anesthetic—Lignocaine)
Lorazepam (Ativan, Temesta)
Meperidine (Demerol, Isonipecaine, Lidol, Pethanol, Piridosal, Algil, Alodan, Centralgin, Dispadol, Dolantin, Mialgin, Petidin, Dolargan, Dolestine, Dolosal, Dolsin, Mefedina)
Methohexital (Methohexitone, Brevital)
Methylprednisolone (a gluco/corticosteroid—Medrol, Solu-Medrol, Cadista)
Metoclopramide (an antiemetic—Maxolon, Reglan, Degan, Maxeran, Primperan, Pylomid, Cerucal, Pramin)
Midazolam (Versed, Dormicum, Hypnovel)
Morphine (MS Contin, MSIR, Avinza, Kadian, Oramorph, Roxanol, Kapanol)
Naloxone (reversal agent for narcotics—Narcan, Nalone, Narcanti)
NalTREXone (Revia, Depade, Vivitrol, Bromide)
Nitrous oxide
Ondansetron (an antiemetic—Zofran)
Pentobarbital (Nembutal)
Propofol (Diprivan)
Remifentanil (Ultiva)
Rocuronium (neuromuscular paralytic)
Scopolamine (levo-duboisine, hyoscine)
Sevoflurane
Succinylcholine (neuromuscular paralytic—Suxamethonium chloride, Suxamethonium, Anectine, Quelicin, Scoline)
Sufentanil (Sufenta)

**Table 2:** Route of sedative administration options from adverse event sedation reporting tool website.

Route of Sedative Administration
Buccal
Inhalation
Intramuscular
Intranasal
Intravenous
Oral
Other
Rectal
Subcutaneous
Sublingual
Topical

**Table 3:** Patient demographic and descriptive data of *n* = 7952 sedation records.

Category	Frequency	Percent
Female	4335	54.9
Male	3569	45.2
<1 year	48	0.6
1–3	279	3.5
3–5	972	12.3
5–12	1050	13.3
12–18	437	5.5
18–50	2734	34.6
50–70	1685	21.3
70–80	527	6.7
≥80	172	2.3
ASA Physical Status		
1	4901	62.0
2	2708	34.3
3	290	3.7
4	4	0.1
5	1	0.0
ASA ≤2 (not emergent)	7470	94.5
ASA >2 (not emergent)	279	3.5
ASA ≤2 (emergent)	139	1.8
ASA >2 (emergent)	16	0. 2

**Table 4:** Frequency of sedations by provider country and place of practice.

Country	Place of Practice	Academic or Non-Academic Setting	Frequency *n* (%)
New Zealand	Non-hospital based	Non-Academic	3328 (42.19)
Australia	Non-hospital based	Non-Academic	1436 (18.20)
UK	Non-hospital based	Non-Academic	958 (12.15)
USA	Non-hospital based	Non-Academic	641 (8.13)
South Korea	Hospital-based	Academic	415 (5.26)
New Zealand	Both	Both	365 (4.63)
USA	Hospital-based	Academic	305 (3.87)
Israel	Hospital-based	Academic	109 (1.38)
Brazil	Non-hospital based	Academic	100 (1.27)
India	Hospital-based	Non-Academic	68 (0.86)
N/A	Hospital-based	Academic	40 (0.51)
India	Hospital-based	Academic	19 (0.24)
UK	Hospital-based	Both	19 (0.24)
UK	Hospital-based	Non-Academic	18 (0.23)
Spain	Hospital-based	Non-Academic	17 (0.22)
Netherlands	Non-hospital based	Non-Academic	10 (0.13)
Italy	Hospital-based	Both	8 (0.10)
Mongolia	Hospital-based	Academic	4 (0.05)
Netherlands	Hospital-based	Non-Academic	4 (0.05)
Belgium	Hospital-based	Both	3 (0.04)
Japan	Hospital-based	Non-Academic	3 (0.04)
South Korea	Hospital-based	Both	3 (0.04)
South Africa	Both	Non-Academic	2 (0.03)
UK	Hospital-based	Academic	2 (0.03)
Australia	Both	Both	1 (0.01)
Chile	Hospital-based	Academic	1 (0.01)
Canada	Hospital-based	Academic	1 (0.01)
India	Hospital-based	Both	1 (0.01)
N/A **	Non-hospital based *	Non-Academic	1 (0.01)
New Zealand	Hospital-based	Both	1 (0.01)
Saudi Arabia	Hospital-based	Both	1 (0.01)
Saudi Arabia	Non-hospital based	Non-Academic	1 (0.01)
UK	Both	Both	1 (0.01)
USA	Hospital-based	Non-Academic	1 (0.01)
USA	Hospital-based	Both	1 (0.01)

* Non-hospital-based place of practice(s) are generally private practice or clinics. ** 41 events had N/A as their country, indicating ‘Not available’. These came from four users for which the country of origin USA.

**Table 5:** Multivariable logistic regression model for adverse events.

Variable	Proportion	*n*/*N*	Odds Ratio	95% CI	*p*-Value
ASA					
I or II	0.006	991/159184	Reference		
III or higher	0.009	46/5345	1.89	1.34–2.67	0.0003
Age (year)					
<1	0.049	39/790	0.99	0.54–1.81	0.9678
1–3	0.020	107/5317	0.60	0.39–0.92	0.0181
3–5	0.006	115/20270	0.33	0.22–0.49	<0.0001
5–12	0.010	203/21104	1.37	0.97–1.94	0.0719
12–18	0.010	86/8895	1.09	0.71–1.66	0.6986
18–50	0.003	173/58458	Reference		
50–70	0.004	158/35507	2.19	1.53–3.13	<0.0001
70–80	0.008	92/10851	2.81	1.83–4.32	<0.0001
≥80	0.019	64/3337	0.82	0.46–1.47	0.5093
Sex					
Female	0.002	184/90883	Reference		
Male	0.003	231/73024	1.13	0.82–1.38	0.2492
Procedure Time					
6:00 am–12:00 pm	0.002	226/114917	Reference		
12:00–6:00 pm	0.003	152/48439	1.40	1.12–1.73	0.0027
6:00 pm–12:00 am	0.076	32/420	8.74	5.43–14.06	<0.0001
12:00–6:00 am	0.038	5/131	5.86	2.24–15.34	0.0003
Procedure Location					
Hospital	0.017	295/17152	Reference		
Non-hospital	0.001	120/146729	0.03	0.02–0.04	<0.0001
Other *	0.000	0/26	<0.001	NE–NE	0.9697

* Other could represent office, clinic, ambulatory-based setting. Reference indicates which group we can compare the other groups against.

**Table 6:** Identification of adverse events by risk descriptors.

	Frequency *n* (%)			
	Sedation Risks	Intervention Risks	Outcome Risks	Severity
Minimal	0	167 (21.7)	0	0
Minor	139 (22.3)	457 (59.5)	385 (93.4)	78 (21.0)
Moderate	389 (62.5)	123 (16.0)	15 (3.6)	228 (61.5)
Sentinel	94 (15.1)	21 (2.7)	12 (2.9)	65 (17.5)
Total	622 (19.06)	768 (19.06)	412 (19.06)	371 (19.06)

**Table 7:** Incidence of adverse events—risks and rates per 10,000.

Risks	Minimal	Minor	Moderate	Sentinel	Total (%)	Rate	95% CI
**Sedation Risks**							
Oxygen desaturation (75–90%) for < 60 s	N/A	0	128	0	128 (20.6)	7.80	6.56–9.27
Airway obstruction	N/A	0	89	0	89 (14.3)	5.42	4.41–6.68
Apnea	N/A	0	53	25	78 (12.5)	4.75	3.81–5.93
Oxygen desaturation, severe (< 75% at any time)	N/A	0	0	63	63 (10.1)	3.84	3.00–4.91
Vomiting/Retching	N/A	47	0	0	47 (7.6)	2.86	2.15–3.81
Failed sedation	N/A	0	30	0	30 (4.8)	1.83	1.28–2.61
Subclinical respiratory depression	N/A	29	0	0	29 (4.7)	1.77	1.23–2.54
Hypertension	N/A	0	26	0	26 (4.2)	1.58	1.08–2.33
Hypotension	N/A	0	23	0	23 (3.7)	1.40	0.93–2.11
Hypersalivation	N/A	22	0	0	22 (3.7)	1.34	0.88–2.04
Tachycardia	N/A	0	19	0	19 (3.1)	1.16	0.74–1.82
Bradycardia	N/A	0	16	0	16 (2.6)	0.97	0.60–1.60
Paradoxical response	N/A	14	0	0	14 (2.3)	0.85	0.51–1.44
Recovery agitation	N/A	11	0	0	11 (1.8)	0.67	0.37–1.21
Prolonged recovery	N/A	6	0	0	6 1.0)	0.37	0.16–0.81
Seizure	N/A	6	0	0	6 (1.0)	0.37	0.16–0.81
Allergic reaction without anaphylaxis	N/A	0	5	0	5 (0.8)	0.30	0.13–0.73
Muscle rigidity, myoclonus	N/A	4	0	0	4 (0.6)	0.24	0.09–0.65
Cardiac arrest/absent pulse	N/A	0	0	3	3 (0.5)	0.18	0.06–0.57
Cardiovascular collapse/shock	N/A	0	0	3	3 (0.5)	0.18	0.06–0.57
Total Sedation Risks	N/A	139 (22.3%)	389 (62.5%)	94 (15.1%)	622		
**Intervention Risks**							
Airway repositioning	0	183	0	0	183 (23.8)		
Supplemental oxygen, new or increased	0	147	0	0	147 (19.1)		
Tactile stimulation or the administration of	0	120	0	0	120 (15.6)		
No intervention performed	114	0	0	0	114 (14.8)		
Oral/nasal airway	0	0	51	0	51 (6.6)		
Bag valve mask assisted ventilation	0	0	49	0	49 (6.4)		
Additional sedative(s)	24	0	0	0	24 (3.1)		
Anti-emetic	22	0	0	0	22 (2.9)		
Rapid IV fluids	0	0	10	0	10 (1.3)		
Antihistamine	7	0	0	0	7 (0.9)		
Antisialogogue	0	7	0	0	7 (0.9)		
Laryngeal mask airway	0	0	6	0	6 (0.8)		
Pressor/epinephrine	0	0	0	6	6 (0.8)		
Reversal agents	0	0	6	0	6 (0.8)		
Chest compressions	0	0	0	5	5 (0.7)		
Tracheal intubation or the administration of	0	0	0	5	5 (0.7)		
Atropine to treat bradycardia	0	0	0	4	4 (0.5)		
CPAP	0	0	1	0	1 (0.1)		
Neuromuscular blockade	0	0	1	0	1 (0.1)		
Total Intervention Risks	167 (21.7%)	457 (59.5%)	124 (16.1%)	20 (2.6%)	768		

**Table 8:** Administration of sedatives.

Sedative Name	Percent
Propofol alone	18.74
Other *	13.76
Fentanyl w/one other drug	11.86
Alfentanil w/one other drug	11.03
Ketamine alone	8.78
Ketamine w/≥2 other drugs	7.59
Ketamine w/one other drug	7.12
Propofol w/one other drug	5.22
Fentanyl w/≥2 other drugs	3.32
Midazolam alone	3.32
Dexmedetomidine w/one other drug	2.14
Midazolam w/one other drug	1.30
Clonidine alone	1.07
Alfentanil w/≥2 or more drugs	0.83
Midazolam w/≥2 other drugs	0.83
Chloral hydrate alone	0.71
Chloral hydrate w/one other drug	0.71
Fentanyl alone	0.71
Alfentanil alone	0.36
Dexmedetomidine w/≥2 other drugs	0.36
Chloral hydrate w/≥2 other drugs	0.24
Clonidine w/one other drug	0.00
Clonidine w/≥2 other drugs	0.00
Dexmedetomidine alone	0.00
Propofol w/≥2 other drugs	0.00

* Other represents the following drugs alone and in combination with other drugs: Albuterol, Atropine, Dexamethasone, Diazepam, Diphenhydramine, Etomidate, Flumazenil, Fosphenytoin, Fospropofol, Meperidine, Morphine, Naloxone, Nitrous oxide, Remifentanil, Scopolamine, Sevoflurane, Sufentanil.
